# Co‐Delivery of Ca‐MOF and Mg‐MOF Using Nanoengineered Hydrogels to Promote In Situ Mineralization and Bone Defect Repair: In Vitro and In Vivo Analysis

**DOI:** 10.1002/adhm.202502630

**Published:** 2025-09-01

**Authors:** Cho‐E Choi, Chao Liang, Yasmeen Shamiya, Sang Jin Lee, Arghya Paul

**Affiliations:** ^1^ Department of Chemical and Biochemical Engineering The University of Western Ontario London ON N6A 5B9 Canada; ^2^ Biofunctional Materials Division of Applied Oral Sciences and Community Dental Care Faculty of Dentistry The University of Hong Kong Sai Ying Pun Hong Kong SAR 999077 China; ^3^ Department of Chemistry The University of Western Ontario London ON N6A 5B9 Canada; ^4^ College of Dentistry Kyung Hee University 26 Kyungheedae‐ro Dongdaemun‐gu Seoul 02447 Republic of Korea; ^5^ Department of Chemical and Biochemical Engineering School of Biomedical Engineering Department of Chemistry The Centre for Advanced Materials and Biomaterials Research The University of Western Ontario London ON N6A 5B9 Canada

**Keywords:** bone repair, injectable and sprayable hydrogel, metal–organic frameworks, nanomedicine, nanoparticle–hydrogel hybrid

## Abstract

Severe bone defects resulting from traumatic injuries or infections are severe skeletal deficiencies that are unable to regenerate on their own. Despite their effectiveness, current treatments including allografts and artificial bone substitutes, have several drawbacks. This includes poor osseointegration, low biocompatibility and biodegradability, limited cell infiltration, and adverse side effects arising from drug‐loaded substitutes. To overcome these challenges, mineral‐based metal–organic frameworks (MOFs) nanoparticles are successfully synthesized and incorporated into polymeric hydrogels to promote bone healing. The study demonstrates that the combination of Ca‐MOF and Mg‐MOF (Ca/Mg‐MOF) nanoparticles, when incorporated into a hydrogel scaffold, can take various forms: sprayable, injectable, and coating material for orthopedic implants. Furthermore, nanoengineered hydrogels significantly enhance osteogenic differentiation and mineral deposition of preosteoblast cells compared to control groups and individual MOFs. This osteogenic property can be attributed to the cumulative release of Ca^2+^ and Mg^2+^ that reached 62.89% ± 3.05 and 18.60% ± 0.65 by day 8, respectively. Micro‐computed tomography and histological analysis of rat model with critical‐size bone defects demonstrate that the bioactive hydrogel can significantly improve new bone formation without using any supplemental drug molecules. These findings underscore the clinical significance of nanoengineered mineral‐based hydrogels to promote osteogenesis and accelerate bone healing.

## Graphical abstract

Metal‐organic frameworks (MOFs) have been synthesized using calcium (Ca‐MOF), magnesium (Mg‐MOF), and as hybrids (Ca/Mg‐MOF) for bone healing applications. MOFs are integrated into hydrogel polymer networks for injectable, sprayable, and coating applications. This hybrid hydrogel platform enhances bone regeneration in rat calvarial defects through synergistic mineral release impacting Wnt and MAPK pathways.

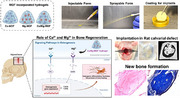

## 1 Introduction

The healing of bones is a crucial biological process necessitating the repair and restoration of injured or fractured bones to their original form and function. This process holds significant importance beyond individual well‐being, extending into the realm of public health. Effective bone regeneration can reduce the costs of hospitalization, rehabilitation, and disability associated with musculoskeletal disorders, alleviating the burden on our healthcare systems, and improving patient outcomes.^[^ ^1^ ^]^ Several human conditions, such as trauma, 3 disease, or aging, may impair the normal function of bones and thus require effective healing.^[^ ^2^ ^]^ Bones are living tissues that can regenerate themselves through various molecular and cellular interactions.^[^ ^3^ ^]^ However, this regeneration process can sometimes be incomplete or delayed due to various factors such as inadequate nutrition, and complexity of the fracture particularly at the craniofacial regions.^[^ ^2^, ^3^, ^4^ ^]^ Management of bone fractures is a considerable challenge in orthopedics. Conventional treatments that have been developed for bone healing typically involve a combination of surgical procedures, casting for immobilization, external metallic fixators, bone grafts, and synthetic implants. These methods aim to provide structural support and promote bone regeneration.^[^ ^5^, ^6^ ^]^ However, there are several drawbacks associated with current bone healing treatment methods, including delayed or non‐union fractures, implant failure, donor site morbidity, immune rejection of the graft, all leading to inadequate bone regeneration.^[^ ^7^, ^8^ ^]^ In 2022, it was reported that although useful for fracture repairs, allografts can result in delayed union or non‐union fractures in 10–30% of cases, depending on varying factors such as patient characteristics, graft type, and surgical techniques.^[^ ^9^, ^10^ ^]^ As a consequence, several other therapies, such as induced membrane technique (IMT) and growth factor‐based therapies have been explored. However, these therapies require additional surgeries, further increasing the risk of infections and/or immune responses, as in the case of IMT, or result in protein instability, rapid diffusion, and off‐target effects, as in the case of growth factor‐based therapies.^[^ ^11^, ^12^, ^13^ ^]^ Inorganic nanoparticles, specifically mineral‐based nanoparticles are promising biomaterials to address these challenges as they are essential to support the various processes involved in natural bone healing.^[^ ^14^, ^15^ ^]^ Minerals play a vital role in the complex biological functions of the living body, particularly in bone biology and the regeneration processes.^[^ ^14^ ^]^ Therefore, the supplementation of minerals presents a promising approach to significantly improve bone healing outcomes compared to alternative treatments.^[^ ^16^ ^]^ Specifically, calcium (Ca^2+^) and magnesium (Mg^2+^) ions hold promise for bone healing applications as they have demonstrated excellent osteoconductivity.^[^ ^17^ ^]^ Calcium is a major component of hydroxyapatite, a mineral that provides strength and hardness to bone tissue. During the bone development process, osteoblast cells, which form bone cells, use calcium to build the mineralized matrix.^[^ ^18^ ^]^ Proper calcium intake is essential for maintaining bone density and preventing conditions like osteoporosis.^[^ ^18^ ^]^ Second, magnesium mediates the process of bone mineralization and has the ability to regulate activity of osteoblasts.^[^ ^19^ ^]^ Besides this, both calcium and magnesium deficiency may result in impaired bone integrity and strength.^[^ ^20^ ^]^ Furthermore, mineral supplementation has been shown to reduce the risk of immunological rejection following transplant surgery, aiding in further improvement of clinical outcomes.^[^ ^21^, ^22^ ^]^ This immunomodulatory effect is complemented by the promotion of new bone tissue formation by these mineral ions, enhancing overall bone strength and stability.^[^ ^23^, ^24^ ^]^

To date, very limited research has been conducted on the effects of co‐delivering Ca^2^⁺ and Mg^2^⁺ for bone regeneration, despite the known positive effects of Mg^2+^ and Ca^2+^ in bone healing.^[^ ^25^ ^]^ However, high concentrations of Ca^2^⁺ and Mg^2^⁺ ions for bone healing may cause toxicity, impaired bone mineralization, disrupted ionic balance, and elicit inflammation, ultimately delaying the healing process.^[^ ^25^ ^]^ Therefore, maintaining optimal ion concentrations is crucial to promote effective bone regeneration, overall physiological balance, normal metabolism, and prevention of bone loss and abnormal biological processes. To address the challenges related with using high concentrations of mineral ions and acknowledge their importance in bone healing processes, metal–organic frameworks (MOFs) can offer promising potential as mineral carriers owing to their distinctive structure and properties.^[^ ^15^, ^24^ ^]^ Comprised of mineral ions and organic ligands, MOFs form a porous crystalline framework with high surface area and adjustable pore sizes.^[^ ^15^, ^24^ ^]^ In the design of MOFs for bone regeneration, the choice of organic ligands is crucial, as it influences structural stability, degradation, and controlled ion release. In this study, trimesic acid (TMA) was selected as the organic ligand for Ca‐MOF and Mg‐MOF due to its high stability, biocompatibility, and ability to regulate the sustained release of Ca^2^⁺ and Mg^2^⁺.^[^ ^26^, ^27^ ^]^ The presence of three carboxyl functional groups allowed strong metal coordination, ensuring controlled degradation and prolonged bioactivity.^[^ ^28^ ^]^ Moreover, MOFs can be tailored to have specific properties, including biocompatibility, biodegradability, making them suitable for biomedical applications.^[^ ^15^ ^]^ With their unique structures, MOFs allows for sustained release of the mineral ions over time.^[^ ^15^ ^]^ This is particularly useful for the release of Ca^2+^ and Mg^2+^ ions for bone healing, as it mimics the natural bone microenvironment, thereby stimulating bone cell activity. These ions are essential for bone healing and their sustained release creates conditions similar to the natural bone environment, thereby stimulating bone cell activity.^[^ ^17^ ^]^ To deliver these therapeutic mineral ions, polymeric hydrogels can be promising vehicles as they can encapsulate MOF nanoparticles. The combined release of both Ca^2+^ and Mg^2+^ ions from the polymeric hydrogels can have a synergistic effect, enhancing the overall bone healing process and accelerating the process of bone healing than either ion alone. Polymeric hydrogels are hydrophilic networks with cross‐links that maintain their 3D structure,^[^ ^29^ ^]^ making them similar to biological tissues and ideal for biomedical applications.^[^ ^30^ ^]^ Their ability to retain biological fluids makes them suitable for drug delivery and wound healing, providing advantages such as the incorporation and sustained release of therapeutic contents.^[^ ^30^ ^]^ Harnessing these properties, a novel approach can be extending their applications to in situ injectable, sprayable, and coating techniques for orthopedic implants (e.g., titanium disk). This approach incorporates the use of a polymeric hydrogel, cross‐linkable gelatin methacrylate (GelMA) hydrogel, as a carrier of therapeutic agents. This polymeric GelMA hydrogel possesses the unique ability to undergo rapid in situ gel‐formation at the targeted defect site under visible‐light irradiation, adapting to various geometrical shapes.^[^ ^15^ ^]^ Its enhanced biological functionalities, due to its biocompatibility and resemblance to the extracellular matrix, further support its effectiveness of bone healing.^[^ ^15^, ^31^ ^]^

This study investigates the therapeutic effects on bone healing, focusing on minimally invasive procedures, enhancement of osteogenic differentiation, and optimization for high‐performance bone healing applications. Additionally, the incorporation of Ca/Mg‐MOFs into the polymeric GelMA hydrogel enables it to serve as an effective vehicle for therapeutic bioactive ions.^[^ ^32^ ^]^ These can be particularly advantageous in procedures such as bone grafts and fracture repair systems, where robust bone healing and integration are essential for successful outcomes. Therefore, we investigated how their incorporation into bone treatments enhances the efficacy of these interventions, ultimately leading to better patient outcomes and improved quality of life. In this study, we investigated the potential of Ca/Mg‐based MOFs incorporated hydrogel for localized bone healing. Our findings demonstrate that this innovative bioactive hydrogel promotes direct interaction with preosteoclast cells, leading to synergistic release of Ca^2+^ and Mg^2+^ ions from the 3D structure, thereby facilitating enhanced bone healing without the use of any supplementary pharmaceutical agents. Our innovative approach presents a robust technique for constructing microporous mineral‐based nanoengineered hydrogel scaffolds, characterized by exceptional biocompatibility and osteoinductivity, which demonstrate considerable potential for clinical applications in bone repair and regeneration.

## 2 Results and Discussion

### 2.1 Synthesis and Chemical Characterization of Ca‐MOF and Mg‐MOF Nanoparticles

**Figure 1:**
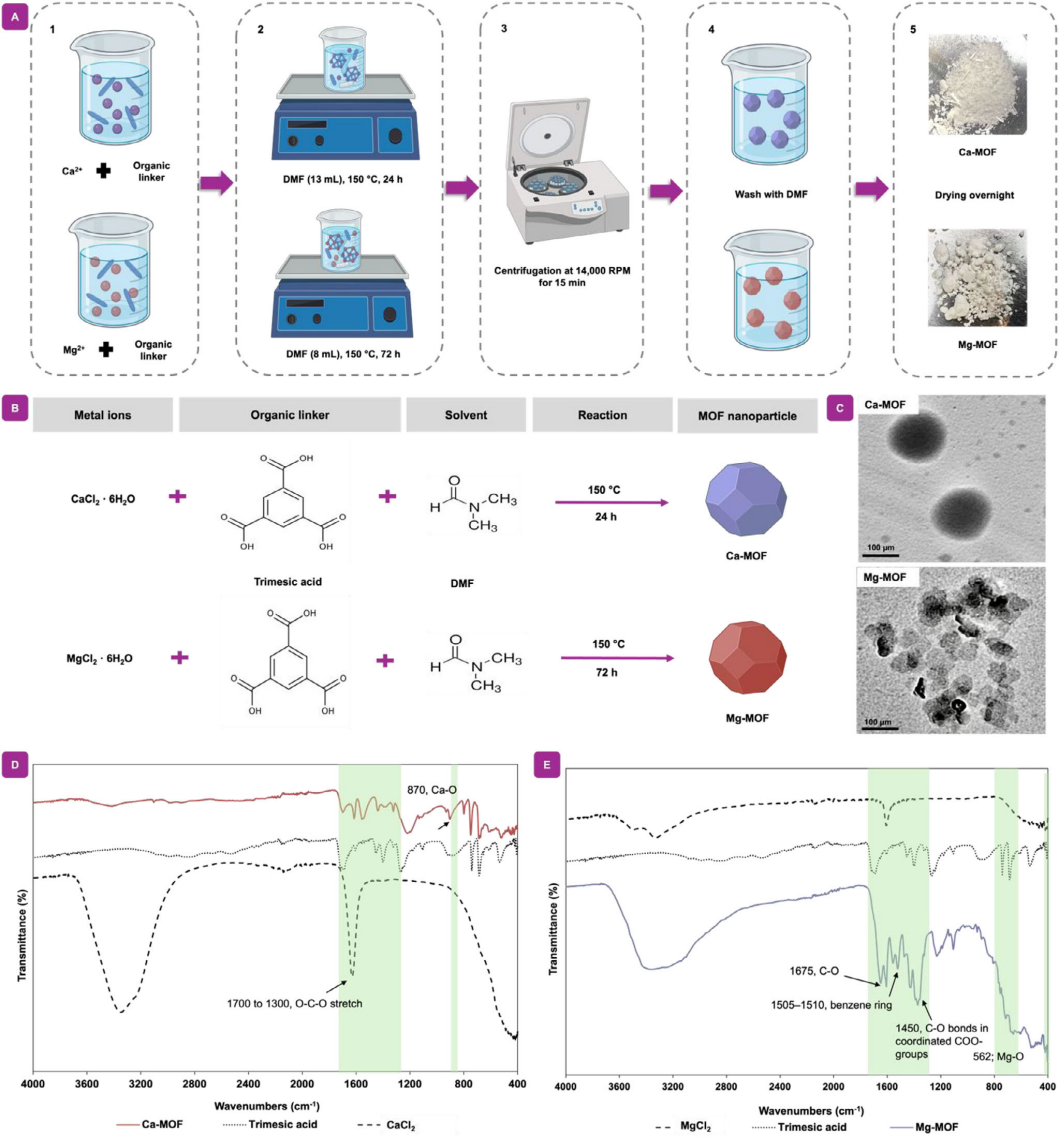
Synthesis and chemical characterization of MOF nanoparticles. A) Schematic detailing the formation of Ca‐MOF and Mg‐MOF nanoparticles. First, Ca and Mg ions are mixed with organic ligands in a DMF solvent under varying conditions. Afterward, the mixtures undergo centrifugation with wash steps to remove unreacted starting materials or by‐products. Finally, the precipitated MOF nanoparticles are obtained and dried for further use. B) Proposed reaction scheme of the synthesis of MOF nanoparticles. C) TEM images of Ca‐MOF and Mg‐MOF nanoparticles: the Ca‐MOF nanoparticles are in a circular form with revealing diameters of ≈100 nm, and the Mg‐MOF nanoparticles are characterized with a diameter of ≈50 nm with a consistent structure. D,E) FTIR spectra of synthesized Ca‐MOF and Mg‐MOF nanoparticles alongside the starting components, respectively.

Ca‐MOF and Mg‐MOF nanoparticles were synthesized utilizing Ca and Mg as the main metal centers, coordinated with an organic ligand to form metal–organic frameworks, as shown in **Figure** **1**A. The synthesis process entails a reaction between either calcium or magnesium ions with organic ligands in DMF as a solvent, where the deprotonation of the organic ligands and subsequent coordination with metal ions facilitate the formation of the MOF backbone.^[^ ^33^ ^]^ Coordination reactions between these ions and trimesic acid as organic ligands drive the self‐assembly of a 3D network structure under stirring conditions, facilitated by the solvent and reaction environment. Small clusters are first nucleated and grown through the continuous addition of metal ions and organic ligands, finally leading to the formation of MOF nanoparticles. After synthesis, Ca‐MOF and Mg‐MOF nanoparticles are isolated and purified to remove any remaining material or by‐products. Figure 1B presents the chemical reaction scheme for the synthesis of MOF nanoparticles, highlighting the coordination between metal ions and the organic ligand.

The morphology and size of Ca‐MOF and Mg‐MOF nanoparticles were characterized by TEM. TEM images for Ca‐MOFs showed a spherical morphology with a diameter of ≈100 nm. On the other hand, Mg‐MOF had a diameter of ≈50 nm and a uniform, circular structure, as shown in the Figure 1C. Here, the organic ligand, trimesic acid, is a precursor compound in this formation. The Ca ions act as the nodes in the MOF structure and control the stability of the framework. Then, the trimesic acid were able to coordinate with the Ca^2+^ and Mg^2+^ ions and form strong bonds, typically through coordinate bonds. The trimesic acid as ligands bind the metal ions at the ends to form the MOF structure.

Figure 1D shows the FTIR spectra of the synthesized Ca‐MOF nanoparticles along with precursor compounds, such as trimesic acid as the organic ligand‐linker, and calcium chloride hexahydrate as the source of Ca ions, thus indicated successful synthesis. In the spectral range of 1700 to 1300 cm^−1^, characteristic peaks belong to the asymmetric and symmetric stretching vibrations of the carboxylic acid (O─C─O). Peaks at 1574.5, 1556, and 1510 cm^−1^ indicate the asymmetric O─C─O stretching, while those at 1435.5 and 1392 cm^−1^ indicate the symmetric O─C─O stretching vibration.^[^ ^34^ ^]^ FTIR analysis of Mg‐MOF nanoparticles in Figure 1E, the sample shows various functional groups with unique adsorption peak at 1679 cm^−1^ indicates characteristic C═O bonds of the carboxylic acids (COOH). Further, the vibrational peak observed at 1505–1510 cm^−1^ is due to the vibration of the benzene ring within the structure. Finally, the Mg─O bond is confirmed with the peak observed at 562 cm^−1^, consistent with reported analyses.^[^ ^35^ ^]^ Next, we optimized a detailed analysis of their physicochemical properties to better understand their chemical composition and physical properties.

### 2.2 Physicochemical Characterization of MOF Nanoparticles and Hydrogel Composites

**Figure 2:**
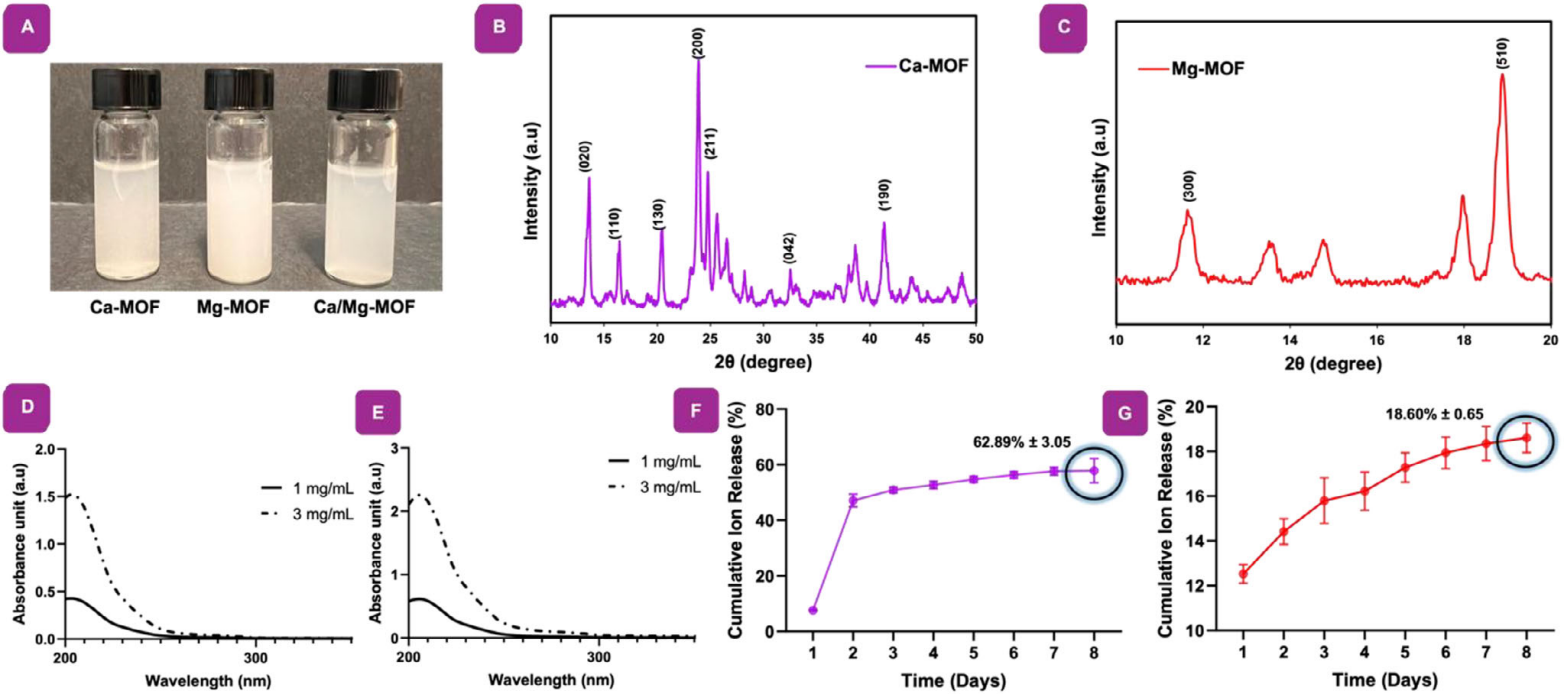
Chemical characterization of synthesized MOF nanoparticles. A) A visual observation of aqueous dispersibility of Ca‐MOF, Mg‐MOF, and Ca/Mg‐MOF nanoparticles. Equal volumes of Ca‐MOF and Mg‐MOF were mixed to obtain a solution of Ca/Mg‐MOF nanoparticles. B) XRD pattern of Ca‐MOF and C) Mg‐MOF describes sharp and robust diffraction peaks, which suggests the crystallinity and crystal structure. D) UV–vis spectra of Ca‐MOF and E) Mg‐MOF, indicating maximum absorbance peaks at 205 and 204 nm, respectively. F) The cumulative ion release of Ca and G) Mg ions demonstrate the cumulative amounts from MOF nanoparticles, confirming that both types of MOF nanoparticles have the ability to sustain ion release (*n = 3*). Each displayed point represents the mean with error bars representing S.D.

MOF nanoparticles can be utilized in diverse biomedical applications including drug delivery systems within aqueous environments such as physiological fluids owing to their tuneable structures, capacity to load and delivery therapeutic agents, and biocompatibility.^[^ ^36^ ^]^ To assess the dispersion of Ca‐MOF, Mg‐MOF, and Ca/Mg‐MOF nanoparticles in water, their behavior in an aqueous solution was evaluated (**Figure** **2**A). All synthesized MOF nanoparticles remained well‐dispersed, demonstrating good aqueous dispersibility. This short‐term dispersion is crucial for understanding their initial behavior in aqueous environments, which is important for potential applications in bone tissue engineering. However, further studies on long‐term stability under physiological conditions are necessary to evaluate their material durability and biological functionality.^[^ ^37^ ^]^

Furthermore, XRD analysis was performed to assess the phase purity and crystallinity of the synthesized MOF nanoparticles. The representative XRD pattern displayed characteristic peaks at 2*θ* values of 8.37°, 13.26°, 17.6°, 25.23°, 29.46°, 31.6°, and 39.6°, corresponding to the crystallographic planes (020), (110), (130), (200), (211), (042), and (190), respectively in Ca‐MOF (Figure 2B). The XRD pattern displayed characteristic diffraction peaks consistent with those observed in a previous study, confirming the successful synthesis of Ca‐MOFs with a crystalline structure.^[^ ^38^, ^39^ ^]^ From the XRD peak pattern of Mg‐MOF, shown in Figure 2C, the formation of Mg‐MOF was confirmed by the presence of characteristic peaks at 2*θ* angles of 11.7°, and 18°, corresponding to the crystallographic planes (300), and (510), respectively.^[^ ^40^, ^41^ ^]^

Additionally, the nanoparticles underwent optical characterization to further confirm the formation of MOF nanoparticles. Examination of the UV–vis spectra (Figure 2D,E) showed a rise in UV absorbance proportional to the increasing nanoparticle concentration. Notably, distinct absorbance peaks were observed at 205 nm for Ca‐MOF and 204 nm for Mg‐MOF.

#### 2.2.1 Release of Ions in the form of MOF Nanoparticles Exhibited Sustained Ion Release Behavior

The study of ion release behavior is crucial for biomedical applications, particularly for ensuring biocompatibility and avoiding potential toxicity from ions like calcium and magnesium.^[^ ^42^ ^]^ Information on ion release kinetics helps optimize therapeutic efficiency by promoting cell proliferation, differentiation, and tissue regeneration.^[^ ^15^ ^]^ Additionally, understanding ion release rates and durations is essential for designing delivery systems for controlled and prolonged release of therapeutic agents, making MOF nanoparticles safe and effective for biomedical use.

Figure 2F,G represents the cumulative amount of ion release from Ca‐MOF and Mg‐MOF nanoparticles until reaching a plateau. On day 8, the ion release behavior of Ca and Mg ions from Ca‐MOF and Mg‐MOF was 62.89% ± 3.05 and 18.60% ± 0.65, respectively. These results indicate that MOF nanoparticles degrade in aqueous solutions, such as in culture media or PBS (simulating physiological environments). From this point of view, these results show that Ca‐MOF and Mg‐MOF nanoparticles can be used as the carrier system to introduce therapeutic ions that can accelerate the process of bone tissue regeneration.

This controlled release profile suggests that Ca‐MOF and Mg‐MOF nanoparticles act as effective ion reservoirs, providing a sustained supply of bioactive ions that enhance osteogenic differentiation and accelerate bone tissue regeneration.^[^ ^43^ ^]^ The initial burst release of Ca^2^⁺ supports early‐stage mineralization and cell adhesion, while the sustained release from MOF degradation over time ensures a continuous bioactive effect.^[^ ^44^, ^45^ ^]^ Although Mg^2^⁺ release was below 20% at day 8, such sustained delivery aligns with its known role in maintaining cellular activity and matrix development over time. Furthermore, incorporating Ca/Mg‐MOF nanoparticles into GelMA hydrogel matrices enables controlled spatial and temporal ion release, which may help regulate local ion concentrations and sustain bioactivity at the defect site. These findings support the potential of Ca‐MOF and Mg‐MOF‐based biomaterials as promising platforms for controlled ion delivery in bone tissue engineering applications. However, longer term release kinetic studies are needed to further understand the degradation properties of Ca/Mg‐MOF nanoparticles within the hydrogel. While the present study utilized a fixed Ca/Mg ratio, the potential synergistic effect between the two ions may be further optimized. Future studies should focus on systematically evaluating a range of Ca/Mg ratios to determine the most effective ion balance for maximizing the therapeutic outcomes.

#### 2.2.2 Swelling Profile

In biomedical applications, swelling capacity of hydrogels is a fundamental property of polymeric hydrogel vital for the diffusion of internal cell nutrients and metabolic byproducts.^[^ ^15^ ^]^ To evaluate the impact of Ca/Mg‐MOF nanoparticles on the swelling behavior of the polymerics hydrogel, a swelling profile was collected by immersing Ca/Mg‐MOF incorporated hydrogels and nanoparticle free hydrogels in PBS for 5 h, as depicted in Figure S1A (Supporting Information). After being immersed in PBS for 5 h, both groups led to a plateau in the swelling ratio (%), where nanoparticle free hydrogels demonstrated a swelling ratio of 420 ± 20.72%, while Ca/Mg‐MOF incorporated hydrogels depicted a swelling ratio of 385 ± 23.38% (Figure S1B, Supporting Information). The addition of Ca/Mg‐MOF nanoparticles does not significantly affect the polymeric hydrogel's swelling behavior, which makes it consistent, and predictable, which is a prerequisite for bone tissue applications.

#### 2.2.3 Degradation Profile

Hydrogel degradation behavior can be controlled so that properties are precisely tuned to make them safe and compatible for a wide variety of biomedical applications, including bone tissue engineering. Also, this mechanism facilitates the sustained release of therapeutic agents as the hydrogel degrades; this plays a crucial role in tailoring the mechanical properties and stability of hydrogels, which are appropriate for use in specific applications such as drug delivery or as implantable devices. Thus, a critical analysis of the hydrogel's degradation is warranted for the optimization of performance and efficacy in biomedical applications.

In this study, we have analyzed the effect of the inclusion of Ca‐MOF and Mg‐MOF nanoparticles on the degradation of hydrogel in PBS supplemented with 1% p/s at 37.5 °C over the course of the two weeks. Degradation of the hydrogel was expected to increase with time. Interestingly, the addition of 6 mg mL^−1^ Ca‐MOF and Mg‐MOF nanoparticles seemed to reduce the rate of degradation compared to nanoparticle‐free hydrogels, giving degradation rates of 71.5% ± 10.84 and 58.66% ± 6.54, respectively, after 14 days (Figure S1C, Supporting Information). The addition of nanoparticles can enhance the mechanical properties of hydrogels, potentially improving their resistance to physical stresses that contribute to degradation. This adds to the comprehensiveness of controlling the degradation rates of hydrogel through physical and/or mechanical means and makes them suitable for therapeutic agent delivery approaches.^[^ ^46^ ^]^ This makes them particularly well‐suited for applications that require sustained delivery of therapeutic agents, where continued efficacy is a requirement.

#### 2.2.4 The Stiffness Properties of the Nanocomposite Hydrogel were Comparable to those of GelMA Hydrogel

A stress sweep test was conducted on the Ca/Mg‐MOF incorporating hydrogel in order to investigate the impact of the nanoparticles Ca‐MOF and Mg‐MOF on stiffness properties. The experimental aim is to investigate the effects of the addition of these nanoparticles on the overall mechanical properties of the hydrogel. Based on Figure S1D (Supporting Information), the rigidity of the Ca/Mg‐MOF integrated hydrogel is the same as that of the traditional polymeric hydrogel and, therefore keeps the same level of mechanical performance. The similar rigidity between the Ca/Mg‐MOF integrated hydrogel and traditional polymeric hydrogel indicates that nanoparticle addition does not compromise mechanical performance. The Ca/Mg‐MOF incorporating hydrogel with comparable storage modulus can achieve effective mimicry with the mechanical properties of native tissues, and as such they can be used in various applications of tissue engineering. This implies that the nanocomposite hydrogel holds great potential for many biomedical applications in which mechanical properties are essential, for example, in the reconstruction of native tissues or the delivery of drugs.

### 2.3 Osteogenic Differentiation of Pre‐Osteoblast: Biological Analysis

#### 2.3.1 In Vitro Cytocompatibility of Individual Ca‐MOF and Mg‐MOF Incorporated Hydrogels

**Figure 3:**
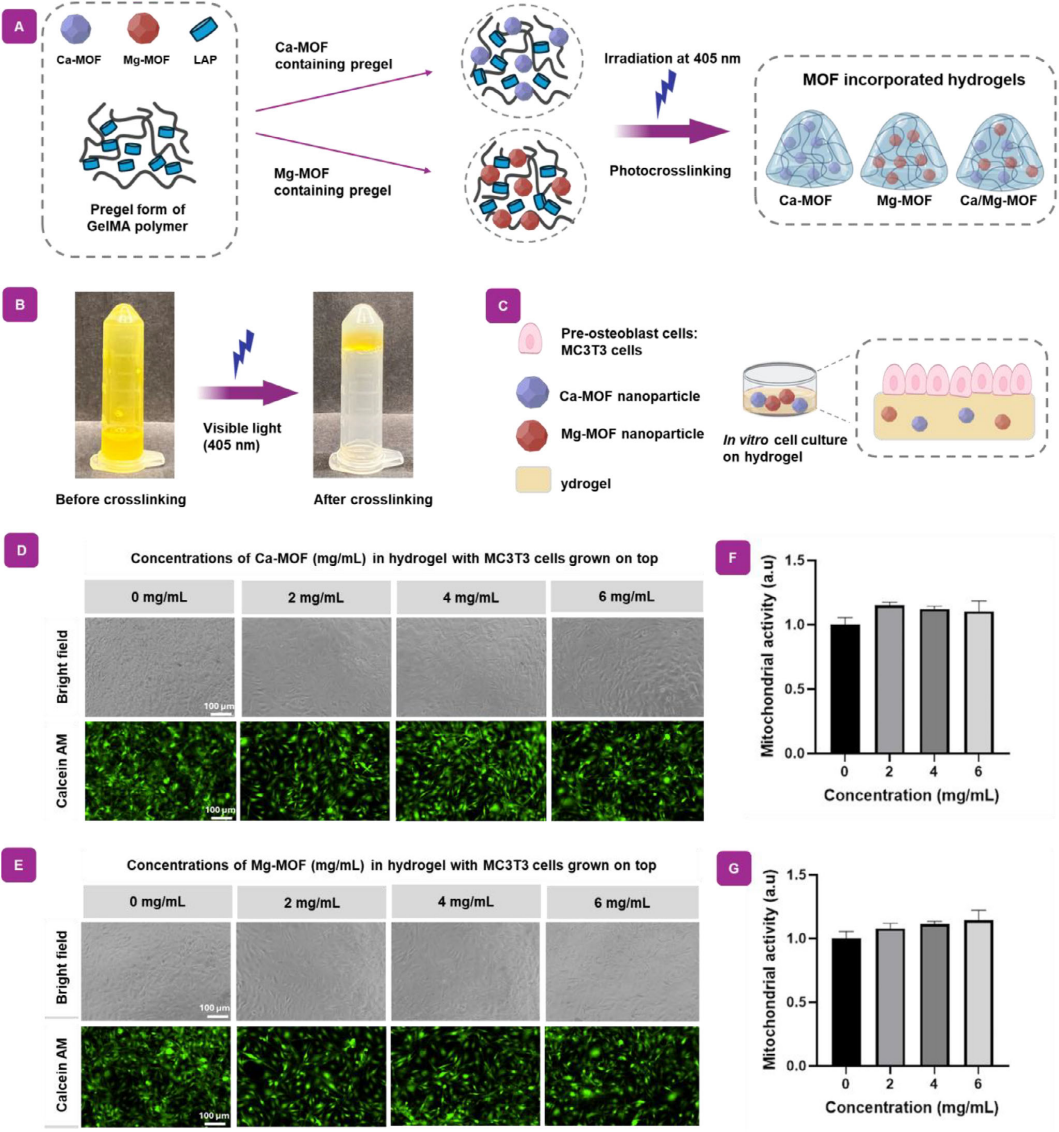
Fabrication and cytocompatibility of in situ injectable Ca‐ and Mg‐MOF nanocomposite hydrogel. A) Nanocomposite hydrogels were fabricated by incorporating Ca‐MOF and Mg‐MOF nanoparticles into hydrogels. The mixture can be then exposed to 405 nm light irradiation in the presence of LAP as a photoinitiator, resulting in a cross‐linked network capable of being injectable. B) Optical images captured before and after cross‐linking, demonstrating the ability of the nanocomposite hydrogel to maintain a stable structure following light irradiation. C) Schematic of the method used to expose the cells to the hydrogel. D,F) Bright field and FITC microscope images reveal MC3T3 cells on Ca‐MOF and Mg‐MOF hydrogels across a range of concentrations from 1 to 6 mg mL^−1^, demonstrating excellent biocompatibility. E,G) Corresponding cell viability was evaluated of Ca‐MOF and Mg‐MOF hydrogel‐treated cells, demonstrating cytocompatibility across a range of concentrations from 1 to 6 mg mL^−1^. Viability levels were comparable to those observed in the 0 mg mL^−1^ control group.

Favorable microenvironments that support cell growth, proliferation, and osteogenic differentiation are important for enhancing bone healing.^[^ ^47^ ^]^ To explore the biological functionalities of individual Ca‐MOF and Mg‐MOF nanoparticles in an in situ injectable application, cross‐linkable polymeric GelMA hydrogels are prepared using these nanoparticles (**Figure** **3**A). Subsequently, the mixture is irradiated with 405 nm light in the presence of a photoinitiator (LAP), yielding a cross‐linked network that is stable after injected sites. Besides, the ability of pregel to undergo gelation indicates that hydrogels containing Ca‐MOF and Mg‐MOF does not impact the ability of the hydrogel to cross‐link (Figure 3B).

To study the biocompatibility of the materials and their interactions with biological conditions, Ca‐MOF and Mg‐MOF containing hydrogels were cultured with pre‐osteoblast cells (MC3T3) at various concentrations to determine the optimal concentration for biocompatibility. This experimental arrangement can be seen in the schematic model of Figure 3C. The cell morphology was observed using bright field microscopy across various concentrations from 0 to 6 mg mL^−1^ of each MOF nanoparticle with hydrogels, while the number of viable cells was observed by Calcein AM cell staining, as shown in Figure 3D,E. Corresponding viable cells in both groups were also studied using MTS analysis, confirming that there is no significant difference in cell proliferation between the control group, which presented MOF free hydrogels, and hydrogels containing up to 6 mg mL^−1^ of Ca‐MOF, Mg‐MOF, or a combination of the two (Figure 3F,G). However, at concentrations above this threshold, a decrease in cell viability was observed, indicating cytotoxic effects at 8 and 10 mg mL^−1^. (Figure S2A,B, Supporting Information). These results indicate that the Ca/Mg‐MOF incorporated hydrogel does not affect cell proliferation rates within this concentration range. Overall, these results demonstrate that the Ca/Mg‐MOF incorporated hydrogel exhibits excellent biocompatibility and effectively supports cell growth, making it a promising candidate for bone tissue regeneration applications. These findings suggest investigating their osteogenic abilities in the next section.

#### 2.3.2 Ca/Mg‐MOF Incorporated Hydrogel Leads to an Increase in Osteogenic Gene Expression and Mineral Deposition

**Figure 4:**
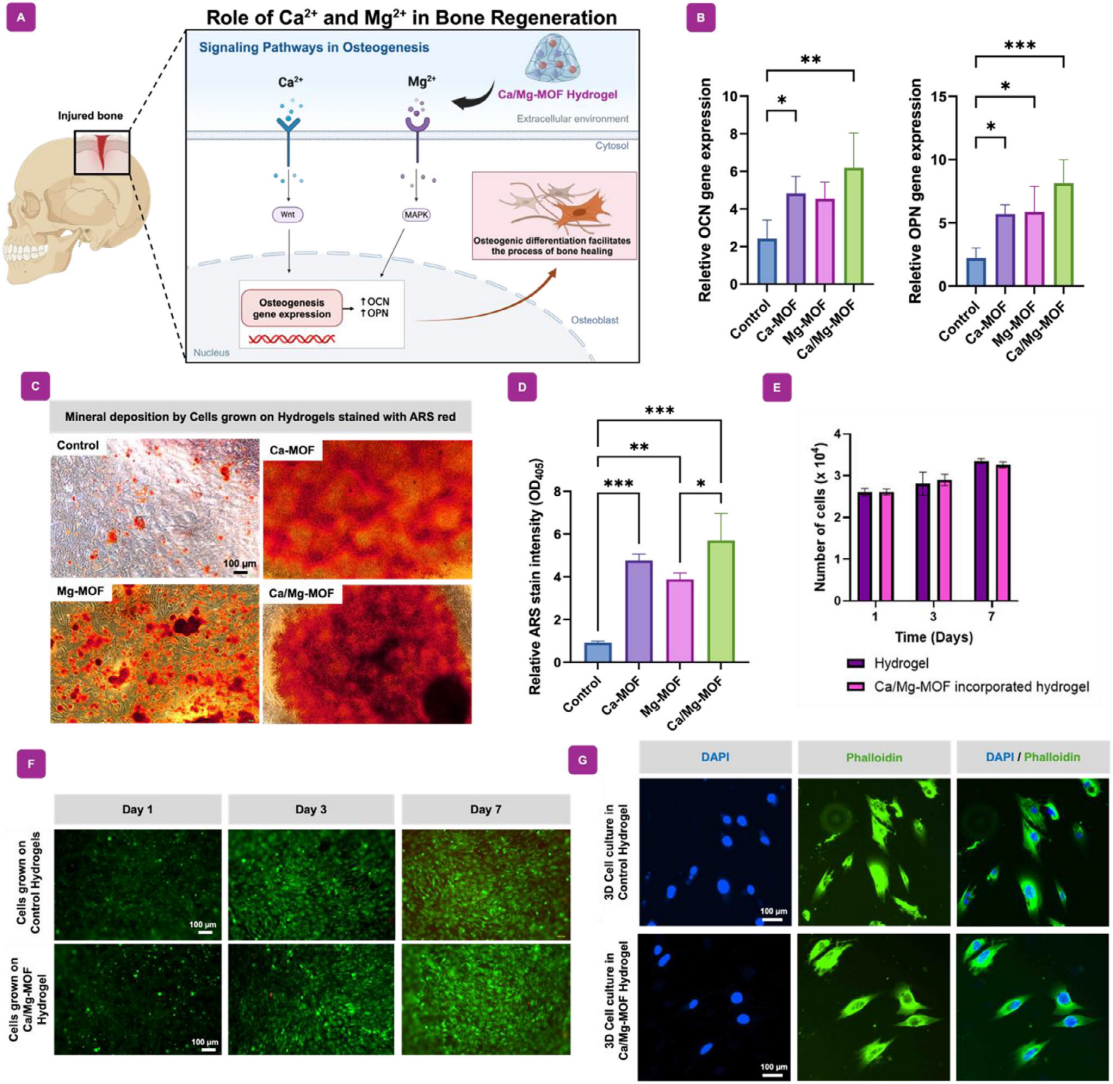
Biofunctionality analysis and osteogenic differentiation of combination Ca/Mg‐MOF incorporated hydrogels. A) The illustration demonstrates the roles of calcium and magnesium ions in the cellular signaling pathways that induce osteogenic differentiation. The degradation of Ca‐MOF and Mg‐MOF nanoparticles into these ions support the bone healing process by inducing osteogenesis gene expression. B) Relative gene expression of osteogenic differentiation, confirming that addition of the MOF nanoparticles further enhanced osteogenic differentiation after 21 days. *p‐value *<*0.05, **p‐value *<*0.01, ***p‐value *<*0.001, and ****p‐value *<*0.0001. C) ARS red stain results revealed high mineral deposition in various MOF nanocomposite hydrogels. D) Quantification of the corresponding ARS stain intensity. E) The number of proliferating cells was analyzed using the MTS assay (*n = 4*) after 24 h of cell culture. F) The biocompatibility of Ca/Mg‐MOF incorporated hydrogels for MC3T3 cells evaluated by Calcein‐AM staining. G) Morphology of cells encapsulated within hydrogels and Ca/Mg‐MOF incorporated hydrogels on day 7. The sample was stained with Phalloidin‐TRITC (red) and 4′,6‐diamidino‐2‐phenylindole or DAPI (blue) and observed by fluorescence microscopy. Cells appeared elongated and well‐spread, which indicates that the cellular microenvironment enabled adhesion and proliferation of the cells. This phenomenon may indicate that these biomaterials might support cell proliferation and the growth of bone tissue. *Abbreviation, PI3K/AKT: Phosphoinositide 3‐Kinase/Protein Kinase B, MAPK: Mitogen‐Activated Protein Kinase, BMP2: Bone Morphogenetic Protein 2, YAP/TAZ: Yes‐Associated Protein/Transcriptional Coactivator with PDZ‐Binding Motif, Wnt: Wingless/Integrated, and β‐Catenin: Beta‐Catenin Signaling.

In the biology of bones, minerals are considered essential elements with critical participation in the process of bone formation, mineralization, growth, and the preservation of bones.^[^ ^17^ ^]^ Specifically, Ca^2^⁺ and Mg^2^⁺ promote osteogenesis through distinct cellular signaling pathways (**Figure** **4**A). Importantly, Mg^2^⁺ influences osteogenic differentiation by modulating integrin‐mediated signaling, particularly through the MAPK pathway and by regulating the activity of alkaline phosphatase (ALP) and other key enzymes involved in bone matrix formation.^[^ ^48^ ^]^ On the other hand, Ca^2^⁺ enhances osteoblast activity and mineralization primarily via activation of the calcium‐sensing receptor, which triggers downstream signaling cascades such as the Wnt pathways, contributing to cell proliferation and matrix mineralization.^[^ ^49^ ^]^ These differing mechanisms allow for a coordinated effect that supports both early and late stages of bone regeneration, therefore we selectively incorporated these ions in the form of MOFs in our hydrogels to test bone repair and regeneration capabilities.

The osteogenic potential of Ca/Mg‐MOF incorporated hydrogels was assessed by analyzing osteogenic gene expression levels using RT‐qPCR. After 21 days of culture, osteogenic gene levels of OPN and OCN, which are common osteogenic bone markers were significantly increased as shown in Figure 4B. Fascinatingly, the hydrogels loaded with Ca/Mg‐MOF nanoparticles showed significantly more expression of genes related to osteogenic differentiation than each individual MOF nanoparticle incorporated hydrogel. These findings indicate the synergistic effect of calcium and magnesium delivery through the hydrogels for effective osteogenic differentiation, which is important in applications toward bone repair. For the assessment of mineral deposition, indicative of advanced osteogenesis, an ARS assay was conducted with MC3T3 cells on day 21. Remarkably, the incorporation of Ca/Mg‐MOF nanoparticles in hydrogels led to a significant enhancement in matrix mineralization in MC3T3 cells compared to the nanoparticle‐free group, as illustrated in the Figure 4C. The associated quantification of ARS staining is depicted in Figure 4D. Overall, these results demonstrate that the delivery of both calcium and magnesium ions in the polymeric hydrogels can produce a synergistic effect on the effective osteogenic differentiation and mineral depositions that is highly crucial for the repair or regeneration of bones.

#### 2.3.3 Cell Morphologies were Investigated in Polymeric Hydrogels

The efficient encapsulation of cells within polymeric hydrogels and further support for their proliferation is essential to biomedical applications.^[^ ^15^ ^]^ Apart from supporting proliferation, the hydrogels need to encapsulate cells efficiently. This is an essential step in attempting to create an environment that boosts cellular activity, proliferation, and ultimately tissue regeneration. By encapsulating cells within the polymeric hydrogel matrix, the hydrogel tries to imitate the native ECM environment, hence giving them structural support and signaling molecules for attachment, spreading, and differentiation.^[^ ^50^ ^]^

Thus, to assess the cell encapsulation capability of the hydrogel, we observed the proliferation of MC3T3 cells encapsulated within the hydrogel over a 24‐h period. Figure 4E reveals that the Ca/Mg‐MOF incorporated hydrogels exhibited a comparable cell proliferation rate to the control groups without Ca/Mg‐MOFs, indicating no detectable toxicity from the nanoparticles. Calcein AM staining was performed to evaluate the biocompatibility of Ca/Mg‐MOF‐incorporated hydrogels by assessing the viability of co‐cultured MC3T3 pre‐osteoblast cells after 1 day. Live cells were stained green, while dead cells were stained red. Fluorescent imaging of live/dead cells within the hydrogels demonstrated sustained cell viability and proliferation over time (Figure 4F). These findings confirm that the incorporation of MOF nanoparticles into hydrogels does not negatively impact cell proliferation when compared to standard hydrogels. Moreover, the analysis of cellular activities within the Ca/Mg‐MOF incorporated hydrogels, in particular cell spreading, revealed quite significant results. After 7 days of culture, the cells encapsulated in both the control and Ca/Mg‐MOF incorporated hydrogels appeared elongated and well‐spread, indicating that the cellular microenvironment facilitated both adhesion and proliferation (Figure 4G). This indicates that the Ca‐Mg‐MOF incorporated hydrogel demonstrates excellent cytocompatibility and is capable of maintaining and supporting cell proliferation, which is the critical prerequisite for successful bone regenerative engineering. It promotes the development of functional and biomimetic tissue constructs equipped for various applications in the domain of regenerative medicine.

### 2.4 The Different Applicability of Ca/Mg‐MOF Incorporated Hydrogel Reveals Promising Potentials for Bone Healing

#### 2.4.1 In Situ Injectable, Sprayable, and Orthopedic Implant Surface Coating with Ca/Mg‐MOF Incorporated Hydrogel, Possibilities, and Opportunities for Biomedical Applications

The fluidity of pregel solutions is very high, which enables smooth application: spraying, injectability, and uniform coating ability for biomedical implant materials.^[^ ^51^ ^]^ Polymeric hydrogels' injectability and sprayability provide significant benefits in biomedical settings. They allow for easy, rapid, and flexible coating over large, complex injury sites, swiftly forming a gel in place.^[^ ^52^ ^]^ The pregel solution is the coating for a variety of biomedical applications such as orthopedic implants (e.g., Ti‐disc). Given that orthopedic implants lack inherent biological functionality and biocompatibility, employing coating approaches can boost their healing process, thereby enhancing their effective treatment.

**Figure 5:**
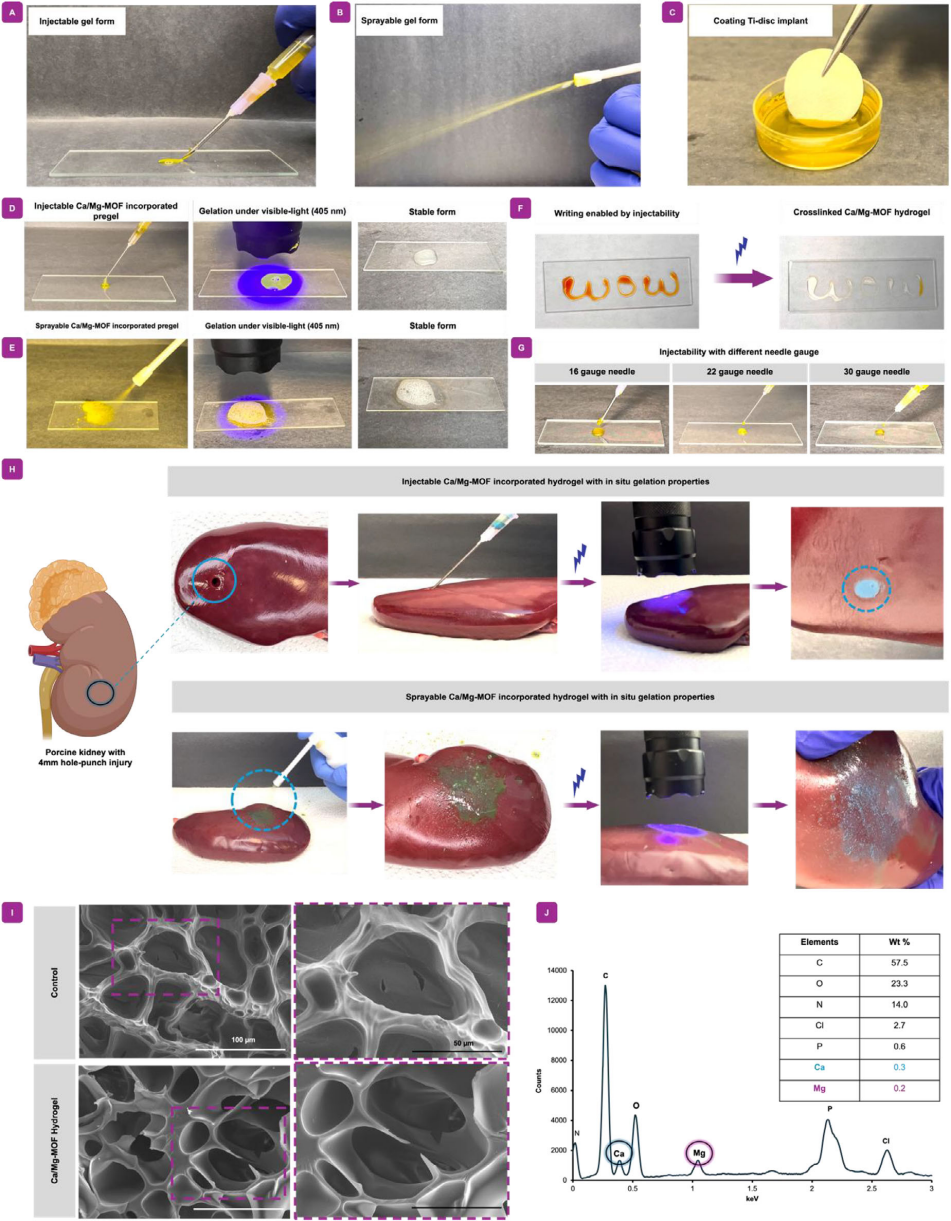
In situ injectable, sprayable, and orthopedic implant coating with cross‐linkable properties of Ca/Mg‐MOF incorporated hydrogel. A) Injecting, B) Spray, and C) Coating titanium disk process of pregel of Ca/Mg‐MOF nanocomposite hydrogel. D) The pregel can be injected and E) sprayed onto the surface of glass, and upon exposure to 405 nm light irradiation for 1 minute, it rapidly and easily forms a stable gelled structure. F) Optical images demonstrate the ability of the pregel solution's injectability to form letters, which can then be cross‐linked to establish a stable hydrogel structure. G) The pregel can be injected using needles of various sizes. H) Injecting and spraying pig kidney submucosa with Ca/Mg‐MOF‐incorporated hydrogel, which contains blue dye, allows for the administration of a pregel. I) SEM images reveal similar porosity in conventional hydrogel and Ca/Mg‐MOF incorporated hydrogel. J) EDX analysis of Ca/Mg‐MOF incorporated hydrogel shows its major elements.

The injectability, sprayable, and coating properties suitable for orthopedic implants, including a titanium disc property of pregel solution of Ca/Mg‐MOF incorporated hydrogels were explored, as shown in **Figure** **5**A–C. To stimulate applicable environments, the Ca/Mg‐MOF incorporated pre‐hydrogel solution was injected and sprayed onto glass surface (Figure 5D,E). When exposed to light (405 nm), the injected and sprayed Ca/Mg‐MOF incorporated hydrogel quickly gelled into a stable structure in under ten minutes. The robust and maintained structures of each group of polymeric hydrogels were demonstrated by optical imaging, which displayed a cylindrical shape generated during cross‐linking (Figure S3A, Supporting Information). The pregel polymer solution was injected using a syringe applicator fitted with a 22 G needle to assess the injectability of the Ca/Mg‐MOF integrated hydrogel. The writing made possible by the syringe applicator was shown using optical images, which also showed how quickly the hydrogel network solidified into the form of letters (Figure 5F).

Furthermore, the cross‐linking properties of the Ca/Mg‐MOF‐incorporated hydrogel were evaluated through a series of experiments to validate the cross‐linking procedure (Figure S3B, Supporting Information). Before cross‐linking, the pregel polymer solution remains in a liquid state but rapidly forms a stable gel structure upon light irradiation (Figure S3C, Supporting Information). Excellent injectability properties of the Ca/Mg‐MOF integrated hydrogel made it simple to extrude through a range of needle diameters frequently employed in medical applications.^[^ ^53^ ^]^ A range of needle gauge sizes, including 16‐, 22‐, and 30‐gauge needles, were evaluated to see how effective they were in assessing injectability. All groups showed their successful injection onto glass surfaces (Figure 5G). To simulate of the applicability of the Ca/Mg‐MOF incorporated hydrogel in animal tissue environments, pork kidney tissues were explored using injection and spraying (Figure 5H). The Ca/Mg MOF‐incorporated hydrogel pregel underwent cross‐linking to form a stable gel on the surface of biological tissue, achieved through both injection and spraying techniques and cross‐linked by visible‐light irradiation. These results suggest that the pregel solution of Ca/Mg‐MOF incorporated hydrogel exhibits favorable cross‐linking behavior in animal tissue environments, making it well‐suited for injection and a promising candidate for bone tissue engineering.

#### 2.4.2 SEM and EDX Analysis Revealed Comparable Porosity of GelMA and its Major Components

Porosity in conventional polymeric hydrogels is one of the vital factors for the viability of cells.^[^ ^54^ ^]^ The porosity within the polymeric hydrogel matrix determines the diffusion of essential substances, vital gases, and metabolic byproducts. Balanced porosity allows an effective supply of nutrients and oxygen to the cells contained in the matrix of the hydrogel, thereby achieving viability and proliferation.^[^ ^54^ ^]^ Therefore, investigation of the interconnected porosity of hydrogels with Ca/Mg‐MOF nanoparticles and samples without nanoparticles was characterized by SEM. (Figure 5I). The observed porosity was found to be comparable between the conventional polymeric hydrogel and the Ca/Mg‐MOF incorporated hydrogel (around 50 µm), which is also important for cell penetration and exchanging nutrients. Moreover, EDX was confirmed to reveal its major elemental component (Figure 5J). The main constituent found in the Ca/Mg‐MOF incorporated hydrogel was composed of carbon, oxygen, and nitrogen. The minor constituent was composed of calcium and magnesium, which showed the presence of both Ca‐MOF and Mg‐MOF nanoparticles in the sample. The main weight percent belonging to it was carbon at 57.5%, followed by oxygen and nitrogen at 23.3% and 14.0%, respectively. The weight percent of calcium and magnesium was recorded at 0.3% and 0.2%, respectively.

#### 2.4.3 In Vivo Characterization of New Bone Tissue Formation After Hydrogel Implantation in Rat Calvarial Defect Model

**Figure 6:**
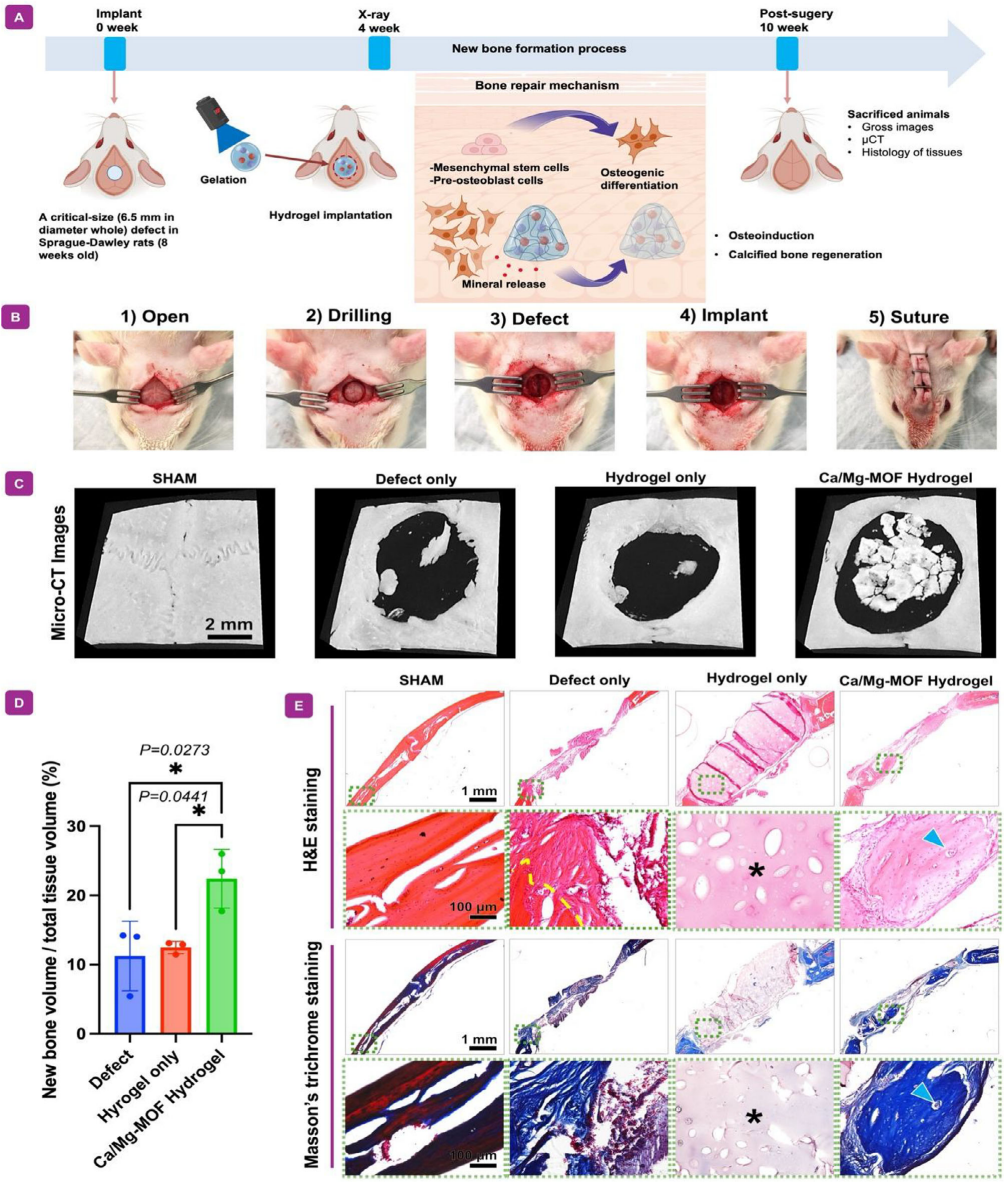
New bone formation after nanocomposite hydrogel implantation in a rat calvarial defect model over 10 weeks. A) A schematic illustration of the in vivo test procedure and the sequential stages of new bone formation, beginning with pro‐osteoblasts, which act as precursor cells that differentiate into mature osteoblasts responsible for bone matrix deposition. Stem cells, as undifferentiated progenitors, have the potential to undergo osteogenic differentiation, a crucial process in which they develop into osteoblasts and contribute to bone tissue regeneration. During this progression, mineral release plays a key role in bone matrix formation, facilitating the deposition of essential minerals that strengthen structural integrity and promote effective skeletal regeneration. B) Surgical steps for creating a standardized rat calvarial bone defect model and hydrogel implantation, from "1" to "5". C) µCT analysis and its corresponding images. D) Quantification of new bone volume within the entire tissue volume. N = 3, *p‐value <0.05. E) H&E and Masson's trichrome staining of transplanted samples at different magnifications. The small black dotted square indicates the location of the high‐magnification image in the low‐magnification histology image. The yellow dotted line marks the original defect border. An asterisk notes the remaining hydrogel within the tissue matrix. Blue arrows denote blood vessels within the newly formed bone.

To verify the potential for new bone tissue formation using the developed hydrogels, a study involving the implantation of a calvarial defect model was conducted. For clear characterization, the rats were randomly assigned to four experimental groups (*n = 3* per group). **Figure** **6**A provides a schematic overview of the 10‐week healing process, highlighting the sequential stages of new bone formation. It is well known that the process begins with pro‐osteoblasts differentiating into mature osteoblasts, which actively deposit bone matrix. The controlled release of minerals from the nanocomposite hydrogel plays a pivotal role in bone matrix formation, reinforcing structural integrity and accelerating skeletal repair.^[^ ^43^ ^]^

In Figure 6B, a critical‐sized calvarial defect was created, followed by the implantation of hydrogels into the defect area. Four weeks post‐surgery, X‐ray imaging was obtained to evaluate the bone formation process (Figure S4, Supporting Information). In the Ca/Mg‐MOF‐incorporated GelMA group, notable filling of the defect was observed compared to the other groups, as indicated by the expansion of the white mineralized area on the X‐rays. This result demonstrated that the Ca/Mg‐MOF nanocomposite hydrogel significantly enhanced the early bone‐like tissue regeneration process in the critical‐sized calvarial defect model. At 10 weeks post‐surgery, all rats were sacrificed for analysis. Optical gross images revealed increased tissue ingrowth, resulting in an opaque appearance in the treated region (Figure S5A, Supporting Information). In all groups, the defect was well filled by new tissue. Notably, the incorporation of Ca/Mg‐MOF into GelMA significantly enhanced tissue repair, showing robust particulates in the central area of the defect. The µCT analysis clearly indicated new bone regeneration within the defect area. In the defect and GelMA groups, a small amount of bone formation was observed. Interestingly, in the Ca/Mg‐MOF‐incorporated GelMA group, substantial calcified bone formed in the center of the defect. This effect may be attributed to the specific properties of the Ca/Mg‐MOF‐hydrogel, including its capacity to release factors (Figure 2) that promote localized cell activity and its structural characteristics that facilitate cell colonization in the defect center. Thus, Ca/Mg‐MOF‐incorporated GelMA group can be engineered to release osteoinductive ions that encourage bone formation. We believe these factors were released more effectively at the center of the wound, this could stimulate bone regeneration from that area. The findings of this result exhibit a pattern similar to previous literatures.^[^ ^55^, ^56^, ^57^ ^]^ The newly formed bone effectively covered the defect, mirroring the tissue coverage seen in the optical images. Importantly, the new bone did not originate from the edges of the defect, indicating that the implanted Ca/Mg‐MOF enabled the formation of new bone. This phenomenon may be attributed to the degradation rate of GelMA, which could hinder tissue ingrowth from the periphery. Instead, the µCT analysis suggested that bone regeneration primarily originated from the periosteum rather than the surrounding bone. These results imply a novel mechanism where the regeneration of critical‐sized bone tissue does not rely solely on adjacent bone but can be initiated from the periosteum. Such findings are promising for the regeneration of large bone defects, as they expand the potential sources for new bone formation.

Quantitative histomorphometric analysis revealed that the bone volume to tissue volume (BV/TV) ratio in the Ca/Mg‐MOF‐GelMA group was nearly double that of the GelMA and defect‐only groups (Figure 6D). These findings confirm that incorporating Ca/Mg‐MOF into GelMA markedly enhances bone regeneration, consistent with the µCT data and underscoring the efficacy of this nanocomposite hydrogel in promoting bone tissue regeneration.^[^ ^58^ ^]^ These results align with in vitro findings on osteogenic induction. Moreover, Ca^2^⁺ and Mg^2^⁺ have been widely shown to support bone regeneration.^[^ ^59^ ^]^ Specifically, Ca^2^⁺ facilitates bone mineralization by regulating the proliferation and differentiation of bone cells through calcium‐sensing receptors, while Mg^2^⁺ upregulates various osteogenic and angiogenic factors and promotes M2 polarization of macrophages.^[^ ^59^, ^60^, ^61^ ^]^

#### 2.4.4 In Vivo Histological Characterization

To assess the pattern of tissue regeneration and confirm bone formation, calvarial defect samples implanted with Ca/Mg‐MOF‐incorporated GelMA and control groups were sectioned and stained with H&E and MT (Figure 6E). At 10 weeks post‐implantation, the Ca/Mg‐MOF‐GelMA group exhibited increased bone tissue ingrowth from the defect border compared to the control and GelMA groups. Although the defect remained partially unfilled, newly generated bone within the border of the original defect area (yellow dotted line) covered most of the new bone with vasculature (blue arrow). Substantial bone regeneration was evident from the border of the defect in the Ca/Mg‐MOF group, narrowing the defect area, which aligned with the µCT findings.

For more detail, the complete and closely magnified histology images of the native host tissue and bone defect are shown in Figure S5B (Supporting Information). The H&E staining revealed the presence of mature bone structures and osteocytes within lacunae, indicating complete bone formation. Furthermore, it showed active osteoblasts adhering to the edge of the new bone, indicating active bone formation^[^ ^62^ ^]^ (red arrow in Figure S5B, Supporting Information). In addition, MT staining was conducted to assess bone maturation. In the Ca/Mg‐MOF‐GelMA group, abundant collagen deposition was evident at 10 weeks post‐implantation, as indicated by the prominent blue staining,^[^ ^63^ ^]^ which was considerably more intense than in the control groups. This enhanced collagen matrix formation implies improved extracellular matrix synthesis and bone tissue maturation facilitated by the Ca/Mg‐MOF nanoparticles. Notably, the residual hydrogel stained a brownish color (asterisks), allowing clear visualization of the original hydrogel placement in both the GelMA and Ca/Mg‐MOF‐GelMA groups. Furthermore, the incorporation of Ca/Mg‐MOF nanoparticles accelerated hydrogel degradation relative to pure GelMA, underscoring the excellent biocompatibility of this novel hydrogel system. This phenomenon is likely attributed to the robust immunomodulation and reactive oxygen species (ROS) scavenging abilities arising from controlled Mg^2^⁺ release, preventing excessive Mg^2^⁺ buildup and hydrogen generation, thereby ensuring successful bone defect healing.^[^ ^64^ ^]^ Moreover, local Mg^2^⁺ accumulation can replicate the natural bone development niche, together with Ca^2^⁺‐triggered angiogenesis through mechanical and biochemical signaling synergistic pathways.^[^ ^65^ ^]^ The synergistic effect promotes endothelial cell migration, thereby inducing neovascularization, which supports the mature vascularization observed in the newly formed bone.^[^ ^66^ ^]^

Therefore, it is believed that the Ca/Mg‐MOF, which can contribute to bone regeneration, was effectively delivered by GelMA. Indeed, GelMA has been utilized in research for the efficient delivery of osteoinductive particles.^[^ ^67^ ^]^ For example, a recent study by Bai et al. explored femoral head necrosis using zinc‐modified MOF and successfully regenerated bone and its repair.^[^ ^68^ ^]^ Similarly, we established a delivery system for the newly developed Ca/MG‐MOF using GelMA, completely promoting bone regeneration. This is expected to significantly contribute to future musculoskeletal tissue engineering and regenerative medicine. Nevertheless, for successful clinical translation, it is essential to evaluate the long‐term stability of the Ca/Mg‐MOF hydrogel system and the biocompatibility of its degradation products under physiological conditions. Future studies should prioritize in vivo assessments to monitor degradation kinetics, potential accumulation of byproducts, systemic toxicity, and inflammatory responses over extended periods. In particular, tuning the hydrogel degradation rate and modifying its composition to better match the rate of new tissue formation will be critical for optimizing integration and therapeutic efficacy. Such evaluations will be vital to fully establish the safety and clinical potential of this new nanocomposite bone healing platform.

### 2.5 Conclusions

In summary, we have developed a nanoengineered hydrogel system using a combination of Ca‐MOF nanoparticles, Mg‐MOF nanoparticles and photo‐cross‐linkable polymers, for treatment of critical‐size bone defects caused by traumatic injuries, bacterial infections or tumor resections. This new biomaterial has the potential to overcome the major challenges encountered by autografts, allografts, bone substitutes and metallic implants, namely donor‐site morbidity, limited bone source, poor biocompatibility and limited osteogenic activity. Herein, we aimed to provide a biomimetic microenvironment to the injury site for accelareted bone healing, which requires persistent local supply of mineral combinations (such Ca^2+^ and Mg^2^⁺), as opposed to individual ions. Here, Mg^2+^ facilitated enhanced osteogenic differentiation of preosteoblats, while Ca^2^⁺ played a crucial role in promoting early‐ and late‐stage mineralization. This makes our hydrogel a promising and safer option for improving bone regeneration. Our hydrogel facilitated controlled release of bioactive Ca and Mg ions, promoting cell adhesion, proliferation, osteogenic differentiation, and mineralization. Their swelling ratio remained comparable to conventional hydrogels, ensuring structural stability while allowing for conformability without inducing excessive tissue strain. Moreover, in vivo experiments with rat calvarial defect model showed increased new bone formation, as evidenced by X‐ray imaging, µCT quantification, and histological analysis. Additionally, the flexibility in preparing this hydrogel allowed us to deliver its precursor to the target site in both injectable and sprayable forms, followed by visible light‐triggered in situ gelation. However, further research is needed to study the effect of this new bioactive hydrogel on oseointegration and vascularization. It is important to note that there are several pro‐osteogenic metal ions such as calcium, magnesium, strontium, zinc, or copper to accelerate new bone formation; however, here, we have chosen Ca^2^⁺ and Mg^2^⁺as a proof of concept to demonstrate the co‐delivery of pro‐osteogenic ions using a nanocomposite hydrogel platform. In future, this combination therapy approach can be applied to other orthopedic conditions that may benefit from the use of our nanoengineered hydrogel system as a regenerative biomaterial, thus making our approach broadly applicable to the treatment other bone‐related disorders such as severe fractures and spinal conditions including degenerative disc diseases.

## 3 Experimental Section

Gelatin from porcine skin was Type A, and methacrylic anhydride was from Sigma–Aldrich, USA. Phosphate‐buffered saline (PBS) was from Fisher Scientific, USA. Zinc nitrate hexahydrate, 2‐methylimidazole, Minimum Essential Medium Eagle with alpha modifications (α‐MEM), fetal bovine serum (FBS), trypsin (0.25%), ß‐glycerophosphate, 2‐methylimidazole (2‐Melm), lithium phenyl‐2,4,6‐trimethylbenzoylphosphinate (LAP), and tartrazine were also purchased from Sigma–Aldrich, USA. Magnesium chloride hexahydrate (cat. BP214500) and calcium chloride hexahydrate (cat. AC38925010) were purchased from Thermo Scientific. Benzene‐1,3,5‐tricarboxylic acid (Trimesic acid, cat. 482749) was purchased from Sigma–Aldrich. N,*N*‐dimethylformamide (DMF, cat. DX1726‐6) was purchased from Supelco. Methanol and EtOH were procured from Fisher Scientific, USA. Penicillin‐Streptomycin (p/s) solution was procured from Gibco, USA.

### Synthesis Metal–Organic Frameworks

*Synthesis of Mg‐MOF Nanoparticles*: Mg‐MOF nanoparticles are synthesized following slightly modified reported method.^[^ ^33^ ^]^ In this process, 4.1 g of magnesium chloride hexahydrate was dissolved in 100 mL of distilled water, and 2.26 g of benzene tricarboxylic acid dissolved in 100 mL of 95% EtOH. A solution of magnesium nitrate was added slowly to the benzene tricarboxylic acid solution. Subsequently, 8 mL of DMF was added, and the mixture was stirred at 150 °C for 3 days in a tightly closed beaker. After washing the precipitate with acetone, the resulting Mg‐MOF was dried in an oven at 60 °C and stored for further analysis.

*Synthesis of Ca‐MOF Nanoparticles*: The Ca‐MOF nanoparticles were synthesized by a slight modification of a previously reported procedure.^[^ ^34^ ^]^ In brief, 0.83 g of calcium chloride hexahydrate was dissolved in 2 mL of deionized water, then 0.63 g of benzene tricarboxylic acid was mixed in 13 mL of DMF and stirred for 60 min. The synthesis was performed at 150 °C for 24 h in a sealed beaker. The synthesized product was precipitated as white compounds and washed well with DMF to remove unreacted compounds. The material was then centrifuged and dried in an oven at 60 °C to evaporate the residual solvent and stored in an airtight tube for further analysis.

### Characterization of MOF Nanoparticles

*Electron Microscopy*: Transmission electron microscopy (TEM) was used in analyzing surface characteristics of synthesized Ca‐MOF and Mg‐MOF nanoparticles through a Philips 420 transmission microscope. Morphology of the synthesized Ca/Mg‐MOF‐based incorporated hydrogels was observed by scanning electron microscopy (SEM) using a Hitachi SU8230 instrument conducted at 20 kV of acceleration voltage. All hydrogels were prepared in a 96‐well plate and were lyophilized at −80 °C for 4 days using a lyophilizer (Labconco, USA). The lyophilized hydrogels were sputter‐coated with a thin layer of gold (6 nm) and imaged. The major composition of samples was also analyzed through energy dispersive X‐ray spectroscopy and elemental mapping using an X‐Flash 6160 EDX detector, Bruker, USA.

*Fourier Transformed Infrared (FTIR)*: FTIR spectra were conducted to determine the chemical composition and functional chemical structure of the samples using a Nicolet Summit FTIR Spectrometer (Thermo Fisher, USA). The FTIR spectra were carried out at room temperature, and the data were obtained in the range of 400 to 4000 cm^−1^.

*UV–vis Spectroscopy*: Optical studies were performed using a UV–vis spectrophotometer (SPARK multimode microplate reader, Tecan, USA) over a wavelength range of 200 – 800 nm to determine the maximum absorbance of Ca‐ and Mg‐based MOF nanoparticles.

*Powdered XRD*: The Ca and Mg‐MOF nanoparticle powdered X‐Ray diffraction analysis (XRD) patterns were conducted using a Bruker Kappa Axis Apex2 (USA).

*Ion Release Kinetic from MOF Nanoparticles*: The ion release profiles of Ca^2^⁺ and Mg^2^⁺ from Ca‐MOF and Mg‐MOF nanoparticles were analyzed using spectrophotometric methods (Abcam, UK; Elabscience, China) following the manufacturers’ protocols. To assess the release behavior, Ca‐MOF and Mg‐MOF nanoparticles (6 mg mL^−1^, *n = 5*) were suspended separately in phosphate‐buffered saline (PBS, pH 7.4) and incubated at 37 °C to simulate physiological conditions. At predetermined time points, the supernatant was collected by centrifugation at 4000 rpm for 5 min, and the concentrations of released Ca^2^⁺ and Mg^2^⁺ ions were quantified via spectrophotometry at 575 and 540 nm, respectively. Standard calibration curves were used to determine the cumulative release of Ca^2^⁺ and Mg^2^⁺ ions over time.

*Dispersion in Aqueous Solution*: Equal volumes (1 mL) and weight (6 mg) of each group were vortexed in water at 25 °C and pH 7.2. The stability of each homogenized group was observed to assess their dispersion.

### Synthesis and Characterization of Ca/Mg‐MOF Incorporated Polymeric Hydrogel

*Preparation of GelMA Pregel Solution*: GelMA was prepared according to previously published methods.^[^ ^69^ ^]^ In an flask, 15% w/v gelatin in PBS was stirred for 1 h at 60 °C until fully dissolved. The dissolved gelatin was further supplemented with 0.8 g mL^−1^ methacrylic anhydride and stirred for 2 more hours at 60 °C. GelMA was then diluted with preheated 100 mL of PBS at 100 °C and dialyzed against distilled water for about one week using a dialysis membrane of 12–14 kDa molecular weight cut‐off. The distilled water was replaced every other day to eliminate the rest of toxic methacrylic anhydride and unreacted components from the GelMA solution. The GelMA was then transferred to 50 mL‐Falcons maintained for 4 days at −80 °C followed by freeze‐drying.

*Preparation of Ca/Mg‐MOF Incorporated Hydrogel*: The pre‐GelMA solution was added to Ca‐MOF and/or Mg‐MOF nanoparticles to prepare Ca/Mg‐MOF incorporated hydrogel formulations with varying concentrations. For individual MOF formulations, Ca‐MOF or Mg‐MOF was added at 0, 2, 4, or 6 mg mL^−1^. For the co‐incorporated hydrogel formulation, Ca‐MOF and Mg‐MOF were both added at 3 mg mL^−1^ each, ensuring a total MOF concentration of 6 mg mL^−1^ for a balanced and controlled ion release. Preparation of cross‐linkable light‐sensitive Ca/Mg‐MOF incorporated hydrogel formulations, LAP (1% w/v) is used as a photoinitiator, and tartrazine (0.1% w/v) was used as a photoabsorber, constant for all formulation. GelMA hydrogel, LAP, tartrazine, Ca/Mg‐MOF nanoparticles, and DI water were mixed and sonicated to ensure adequacy in mixing all components to produce a homogenous mixture of the light sensitive Ca/Mg‐MOF incorporated hydrogel formulation. The final Ca/Mg‐MOF incorporated hydrogel formulation was left in the oven at 60 °C until ready to be used for cross‐linking in the presence of visible‐light at 405 nm.

*Degradation Behavior*: To assess degradation behavior, gelation of GelMA hydrogels (150 µL) as the control group and Ca/Mg‐MOF incorporated hydrogels (*n = 4*) were lyophilized, then their dried weights (*W*₀) were recorded. Subsequently, they were immersed in PBS at 37.5 °C for 0, 1, 3, 7, and 14 days. Following each time interval, the hydrogels were subjected to freeze‐drying again to measure their weights (*W*_D_). The degradation percentage (%) was then determined using the following equation:

Degradation (%)

(1) $$Degradation\left(\%\right)=\frac{W_{o}-W_{D}}{W_{o}}\times 100$$

where *W*_o_ represents the initial weight (mg) of the dried hydrogel, and *W*_D_ is the weight (mg) of the dried hydrogel at specific time points.

*Swelling Behavior*: The swelling ability of dried hydrogels was determined by soaking them in 1 mL of PBS at 37 °C (*n = 4*). Equilibrium swelling was evaluated by measuring their weight of the swollen hydrogels every 30 min up to 5 h. The swelling ratio (%) at each time interval was evaluated by the following equation:

Swelling Ratio (%)

(2) $$Swelling\;Ratio\left(\%\right)=\frac{\left(W_{s}-W_{o}\right)}{W_{o}}\times 100$$

where *W*_S_ represents the weight (mg) of the swollen hydrogel at each time points, and *W*_o_ is the initial weight (mg) of the dried hydrogel.

*Rheological Properties*: Rheological analyses were performed using a HAAKE Modular Advanced Rheometer System (MARS) (Fisher Scientific, USA). Both Ca‐MOF and Mg‐MOF incorporated hydrogels, as well as nanoparticle‐free control hydrogels, were tested using a P20/Ti titanium plate. Stress sweep tests were conducted at a physiological temperature of 37 °C, with the applied stress ranging from 0.1 to 10^2^ Pa.^[^ ^70^ ^]^

### Cytocompatibility and Biofunctionality of Ca/Mg‐MOF Nanocomposite Hydrogels

*In Vitro Cell Culture*: The preosteoblastic cell line MC3T3 (Sigma–Aldrich, USA) originated from C57BL/6 mouse calvaria. and was cultured according to the manufacturer's guidelines. Briefly, MC3T3 cells were incubated at 37 °C in a 5% CO_2_ atmosphere incubator using α‐MEM supplemented with 10% fetal bovine serum (FBS; Millipore Sigma, USA) 1% penicillin/streptomycin (p/s; Millipore Sigma, USA). The medium was replenished every 3 days, and cell harvesting was performed using 0.25% trypsin (Millipore Sigma, USA), followed by resuspension in fresh α‐MEM. Osteogenic differentiation was induced by supplementing α‐MEM with 10% FBS, 1% penicillin/streptomycin (p/s), 100 nm Dexamethasone, and 10 mm ß‐glycerophosphate.

*Cell Proliferation and Cytocompatibility*: Cell proliferation was assessed using 3‐(4,5‐dimethylthiazol‐2‐yl)‐5‐(3‐carboxymethoxyphenyl)‐2‐(4‐sulfophenyl)‐2H‐tetrazolium (MTS; Promega, USA) assay following the manufacturer's instructions. Initially, the Ca/Mg‐MOF incorporated hydrogels were loaded in a 96‐well plate for cross‐linking under visible‐light at 405 nm. Then, MC3T3 cells were cultured onto the hydrogels at a seeding density of 1 × 10^4^ cells per well. The cells were incubated with the hydrogels for 24 h. The culture media was replaced with 20 µL of MTS reagent containing 100 µL of fresh culture media and then the cells were incubated at 37 °C for 2 h. The cell proliferation was evaluated by the absorbance at 490 nm using a 96‐well plate reader (The SPARK multimode microplate reader, Tecan, USA). The morphology of the cells was captured by microscopy (Nikon, Eclipse Ti2, USA).

*Determining Cell Viability of Individual Ca‐MOF and Mg‐MOF Nanoparticles*: The cell viability was determined after incubation with various concentrations of individual Ca‐MOF and Mg‐MOF (0, 2, 4, and 6 mg mL^−1^) incorporated hydrogels. Calcein‐AM (Thermo Fisher, USA) was performed to measure viable cells according to the manufacturer's protocol. In brief, 20 µL of hydrogels were prepared with the different concentrations of single MOF nanoparticles were then added to a 96‐well plate. The pregel was then cross‐linked under visible light irradiation at 405 nm for 10 min. After washing the pregel three times with fresh PBS for 18 h to eliminate the unused components and tartrazine, MC3T3 cells were seeded into the cross‐linked hydrogels at a cell density of 1 × 10^4^ cells per well. After 24 h of culture, 2 µm Calcein AM was prepared in PBS and was then incubated to MC3T3 cells for 20 min in a 37 °C incubator. Lastly, the live cells were visualized using fluorescence microscopy (Nikon, Eclipse Ti2, USA) with excitation wavelength at 495 nm and emission at 515 nm.

*Cells Viability Assessment in Ca/Mg‐MOF Nanocomposite Hydrogels using Live/Dead Cell Staining*: A Live/Dead cell imaging assay (Invitrogen, USA) was used to determine the cell viability of Ca/Mg‐MOF incorporated hydrogels following the manufacturer's guidelines. Initially, MC3T3 cells were seeded onto 0.2 mL of GelMA and Ca/Mg‐MOF incorporated hydrogels at a density of 1 × 10^4^ cells per well. Next, the hydrogels were cultured in fresh culture media at 37 °C in a 5% CO_2_ incubator for 1 day, 3 days, and 7 days of culture. At each time point, the cells were stained using the Live/Dead cell staining kit and visualized using a standard microscope with FITC and Texas Red filters,

*Morphological Analysis of MC3T3 Preosteoblasts on Ca/Mg‐MOF Incorporated Hydrogels*: The morphological analysis of the MC3T3 cells was conducted in the presence of Ca/Mg‐MOF incorporated hydrogels. After 7 days of cell culture, the MC3T3 cells were fixed in 4% formaldehyde for 10 min at room temperature. The cells were then gently washed two times with PBS. Subsequently, the cells were incubated at room temperature for 30 min with Phalloidin‐iFluor 488 Reagent. After, the cell nuclei were stained with DAPI stain (1:5000) of EMD Millipore Corporation, USA, for 15 min and washed with PBS. The stained cells were then observed and captured using a Nikon Eclipse Microscope, Nikon, Canada.

*Assessing Mineral Deposition in Preosteoblasts Through Alizarin Red S (ARS) Staining*: Alizarin red S solution (ARS; ARS Staining Quantification Assay, ScienCell Research Laboratories, USA) was used to determine calcium deposition in the MC3T3 cells. After 21 days of cell culture, 0.5 mL of ARS solution was added to the cells for 30 min at room temperature. Then, the cells were fixed with 4% formaldehyde for 10 min at room temperature and then washed with distilled water to remove excess ARS solution. The ARS‐stained cells were visualized using a Nikon Eclipse Microscope (Nikon, Canada). To quantify the intensity of ARS, 10% acetic acid was applied to the ARS‐stained cells and incubated for 10 min at 85 °C. After incubation, the cells were collected and centrifuged for 15 min at 20 000 g. The supernatant was then transferred and neutralized with 10% ammonium hydroxide. Subsequently, 150 µL of each sample was measured for optical density at 405 nm (OD_405_) using the Spark multimode microplate reader (Tecan, USA).

*Assessing the Osteogenic Differentiation Promotion of Preosteoblast Cells by Ca/Mg‐MOF Incorporated Hydrogels Through Reverse Transcription‐Quantitative Polymerase Chain Reaction (RT‐qPCR) Analysis*: MC3T3 cells were cultured in 24‐well plates in an osteogenic medium for 21 days with the medium replacement every 3 days. Total RNA was extracted using RNeasy Micro Kit (Qiagen, USA) and 150 µg of RNA was used for reverse transcription into cDNA using High‐Capacity cDNA Reverse Transcription Kit (Thermo Fisher Scientific, USA) according to the manufacturer's protocol. Gene expression analysis was conducted using TB Green Advantage qPCR premix (Takara, USA). Targeted genes, including osteopontin (OPN) and osteocalcin (OCN) known regulators of osteoblast differentiation, were quantified. RT‐qPCR analysis was replicated in triplicate for each group. The ΔΔC_T_ method normalized to the reference gene glyceraldehyde 3‐phosphate dehydrogenase (GAPDH) was employed to calculate the expression levels of the target genes. The primer sequences for the targeted genes are provided in **Table** **1**.

**Table 1:** Primer sequences used for RT‐qPCR.

Gene	Forward primer sequence (5′‐3′)	Reverse primer sequence (5′‐3′)	Ref
Murine OCN	AAGCAGGAGGGCAATAAGGT	TTTGTAGGCGGTCTTCAAGC	[71]
Murine OPN	AGCAAGAAACTCTTCCAAGCAA	GTGAGATTCGTCAGATTCATCCG	[72]
Murine GAPDH	AGGTCGGTGTGAACGGATTTG	TGTAGACCATGTAGTTGAGGTCA	[73]

### In Vivo Implantation of a Ca/Mg‐MOF Nanocomposite Encapsulated Hydrogel in a Rat Calvarial Defect Model

Sprague‐Dawley rats (8 weeks old) were used in this study. All animal studies were conducted under the approved animal research protocol (No. 24–026) by the Committee on the Use of Live Animals in Teaching and Research (CULATR) at the University of Hong Kong. The studies adhered to the Animals (Control of Experiments) Ordinance (Hong Kong) and guidelines from the Centre for Comparative Medical Research (CCMR), Li Ka Shing Faculty of Medicine, The University of Hong Kong. Surgical procedures were performed under anesthesia using intraperitoneal injections of 100 mg kg^−1^ Ketamine (Cat. No. 013004, AlfaMedic Ltd.) and 10 mg kg^−1^ Xylazine (Cat. No. 013006, AlfaMedic Ltd.), with Buprenorphine (0.05 mg kg^−1^, ATCvet Code: QN02AE01, Bupaq, Richter Pharma AG, Austria) administered prior to surgery. A midline incision was made over the calvarium, and a full‐thickness flap was elevated. A critical size calvarial defect, round in shape with a diameter of 6.5 mm, was created by drilling a bone defect with a trepan burr under sterile saline irrigation. The detailed procedures described in previous literature.^[^ ^74^ ^]^ The rats were randomly assigned to four experimental groups (*n = 3* per group): 1) Sham; 2) Defect only; 3) GelMA; and 4) GelMA + Ca/Mg‐MOF. After defect creation, the hydrogel was placed over the defect area, and the flap was sutured post‐implantation. Each animal received subcutaneous injections of Buprenorphine twice daily for two consecutive days after surgery. X‐ray images were taken at 4 weeks to examine the surgical area. All animals were euthanized 10 weeks post‐surgery by injecting Dorminal 20% Inj (Cat. No. 013003, Pentobarbital, AlfaMedic Ltd.). After euthanasia, the calvarial bones were carefully extracted, followed by fixation in 10% neutral buffered formalin (NBF) for 48 h. Finally, samples were preserved in 70% EtOH and subsequently subjected to micro computed tomography (µCT) scanning and quantitative analysis.

### Micro‐Computed Tomography Scan Analysis

New bone formation at the defect sites was evaluated using a µCT scanner (Bruker, Skyscan 1276, Germany). The embedded specimens were fixed in a cylindrical sample holder with a diameter of 15.4 mm, positioning the coronal aspect of the calvarial bone in a vertical orientation. The scanning direction of the specimens was perpendicular to the coronal aspect of the calvarial bone. High‐resolution scanning was performed, with an in‐plane pixel size and slice thickness of 13.4 µm, covering the entire thickness of the calvarial bone. The µCT scanner's built‐in software (Bruker CTVol) was used to generate a 3D reconstruction from the set of scans. A cylindrical region of interest (ROI) measuring 6.5 mm in diameter and extending through the full height of the scans was selected from the complete 3D dataset for analysis. Since the edges of the original defect were clearly visible, the ROI was positioned directly at the location of the initial defect.

### Stereo Microscopic and Histologic Evaluations of Tissue Samples

After completing the µCT analysis, the bone samples were transferred to the histology laboratory and stored in 70% EtOH prior to histological analysis. The macroscopic features of each sample were captured using a NIKON SMZ18 stereo microscope (Tokyo, Japan) equipped with a DFK 33UX183 camera (Bremen, Germany) under bright field illumination. For paraffin‐based histology, the samples were decalcified in a 12.5% ethylenediaminetetraacetic acid solution under shaking for 42 days at room temperature. Following decalcification, the samples underwent standard tissue processing for sectioning. Thin cross‐sections, each 14 µm thick, were cut and placed on slides. After paraffin removal and dehydration steps, slides were stained by hematoxylin and eosin (H&E) and Masson's Trichrome (MT). After drying, stained slides were mounted with xylene based mounting medium and then observed using an optical microscope (Cat. No. Eclipse LV100POL, Nikon, Tokyo, Japan) equipped with a CCD camera (Cat. No. DS‐Ri1, Nikon, Tokyo, Japan).

### Statistical Analysis

One‐way ANOVA was used to determine statistical significance for any studies having more than two groups, including MTS and RT‐qPCR experiments on cell proliferation for the analysis of the gene expression profiles and quantification of new bone in animal study. A Tukey procedure was used for post hoc analysis. *p* <0.05 was considered statistically significant.

## Conflict of Interest

The authors declare no conflict of interest.

## Supporting information



Supporting Information

## Data Availability

The data that support the findings of this study are available from the corresponding author upon reasonable request.
