# Supplementary material for: Co‐Delivery of Ca‐MOF and Mg‐MOF Using Nanoengineered Hydrogels to Promote In Situ Mineralization and Bone Defect Repair: In Vitro and In Vivo Analysis
Source: Adv Healthc Mater. 2025 Sep 1;14(32):e02630. doi: 10.1002/adhm.202502630 (PMC12716217; doi:10.1002/adhm.202502630)
Supplement: Supplementary file 1 — Supporting Information [file ADHM-14-0-s001.docx]

Supporting Information

**Co-delivery of Ca-MOF and Mg-MOF using nanoengineered hydrogels to promote *in situ* mineralization and bone defect repair: in vitro and in vivo analysis**

Cho-E Choi#, Chao Liang#, Yasmeen Shamiya, Sang Jin Lee#, and Arghya Paul#*

**Cho-E Choi**

Department of Chemical and Biochemical Engineering, The University of Western Ontario, London, ON N6A 5B9, Canada

**Chao Liang**

Biofunctional Materials, Division of Applied Oral Sciences and Community Dental Care, Faculty of Dentistry, The University of Hong Kong, Sai Ying Pun, Hong Kong Special Administrative Region

**Yasmeen Shamiya**

Department of Chemistry, The University of Western Ontario, London, ON N6A 5B9, Canada

**Sang Jin Lee***

Biofunctional Materials, Division of Applied Oral Sciences and Community Dental Care, Faculty of Dentistry, The University of Hong Kong, Sai Ying Pun, Hong Kong Special Administrative Region

E-mail: dentsj@hku.hk

**Arghya Paul***

Department of Chemical and Biochemical Engineering, School of Biomedical Engineering, Department of Chemistry, The Centre for Advanced Materials and Biomaterials Research, The University of Western Ontario, London, ON N6A 5B9, Canada
E-mail: [arghya.paul@uwo.ca](mailto:arghya.paul@uwo.ca)

**Supplementary Figure 1.** Physiochemical and mechanical characterization of Ca/Mg-MOF hydrogel. A) Optical images of Ca/Mg-MOF hydrogel before and after retaining water for 5 hours. B) The swelling ratio profile of hydrogels and Ca/Mg-MOF hydrogels indicates an expansion to approximately 4.2 times and 3.8 times their original weight, respectively (*n = 5*). C) The degradation profile shows that the addition of nanoparticles reduced the rate of hydrogel degradation after 14 days (*n = 5*). D) The shear stress of Ca/Mg-MOF incorporated hydrogel exhibited a comparable stiffness to that of conventional hydrogels. Each displayed point represents the mean with error bars representing S*.*D.

**Supplementary Figure 2.** A) Fluorescence microscopy images (FITC channel) showing live MC3T3 cells stained with Calcein AM after culture on hydrogels containing Ca-MOF and Mg-MOF at concentrations of 8 mg/mL and 10 mg/mL. Scale bar indicates 100 μm. B) Quantitative analysis of cell viability for MC3T3 cells cultured on Ca-MOF- and Mg-MOF-loaded hydrogels at the indicated concentrations (*n = 3*). In the main manuscript (Figure 3), mitochondrial activity for 0-6 mg/mL has been shown for Ca-MOF and Mg-MOF. Here, we report that at a concentration higher than 6 mg/mL, Ca-MOF and Mg-MOF can potentially be toxic to the cells.

**Supplementary Figure 3.** Representative optical images of crosslinkabled hydrogels. A) Optical images showed each group of polymeric hydrogels displaying a cylindrical structure formed following crosslinking. B) Series images depicted the process of injected pregel undergoing crosslinking to form stable gelation upon exposure to light irradiation (405 nm). C) Pregel initially exhibits a liquid state before crosslinking, whereas the crosslinked gel demonstrates a stable structure.

**Supplementary Figure 4.** Representative X-ray images of each group show the bone volume fractions formed within the defect site four weeks post-surgery.

**Supplementary Figure 5.** A) Representative optical gross images of each group taken 10 weeks post-surgery. B) H&E and Masson’s trichrome staining of transplanted samples at different magnifications (2×, 5×, and 20×). The 2× image provides a full view of the entire defect model. The 5× images show: (i) the left border, (ii) the center, and (iii) the right border of the defect area. Gray dotted rectangles indicate the 20× magnification, displayed in the upper right of the corresponding 5× images. The yellow dotted line marks the original defect border; NB denotes newly formed bone, and the region within the yellow dotted line but adjacent to NB indicates the remaining defect. Red arrows indicate osteoblasts lining the border of the newly formed bone. Blue arrows denote blood vessels. Asterisks indicate remaining hydrogel within the tissue matrix.
